# The Efficiency of Highly Porous β-Tricalcium Phosphate With Bone Marrow Aspirate Concentrate in Midfoot Joint Arthrodesis

**DOI:** 10.1177/19386400231213177

**Published:** 2023-11-29

**Authors:** Wonyong Lee, Dan Prat, Wen Chao, Daniel C. Farber, Carol Wang, Keith L. Wapner

**Affiliations:** Division of Foot & Ankle Surgery, Department of Orthopaedic Surgery, University of Pennsylvania, Philadelphia, Pennsylvania; Department of Orthopaedic Surgery, Guthrie Medical Group, Sayre, Pennsylvania; Division of Foot & Ankle Surgery, Department of Orthopaedic Surgery, University of Pennsylvania, Philadelphia, Pennsylvania; Division of Foot & Ankle Surgery, Department of Orthopaedic Surgery, University of Pennsylvania, Philadelphia, Pennsylvania; Division of Foot & Ankle Surgery, Department of Orthopaedic Surgery, University of Pennsylvania, Philadelphia, Pennsylvania; Perelman School of Medicine, University of Pennsylvania, Philadelphia, Pennsylvania; Division of Foot & Ankle Surgery, Department of Orthopaedic Surgery, University of Pennsylvania, Philadelphia, Pennsylvania

**Keywords:** midfoot, arthrodesis, nonunion, bone substitute, bone marrow aspirate concentrate

## Abstract

**Background:**

Nonunion is one of the most common and devastating complications following midfoot joint arthrodesis. Many different types of bone grafts and bone substitutes have been used to promote osseous fusion. However, there is no consensus on the gold standard bone grafting material and whether biologic materials should be used alone or in combination. The purpose of this study is to investigate the efficiency of highly porous β-tricalcium phosphate (β-TCP) with bone marrow aspirate concentrate (BMAC) in midfoot joint arthrodesis.

**Methods:**

This retrospective comparative study included patients who underwent midfoot joint arthrodesis using compression screws. Patients were classified into 2 groups: arthrodesis with highly porous β-TCP and BMAC (group A) and arthrodesis without them (group B). The osseous union rate was compared between the 2 groups. A total of 44 patients (46 feet) including 89 joints were included in this study.

**Results:**

There was a significant difference in the union rate between the 2 groups: 91.5% (43/47 joints) in arthrodesis with highly porous β-TCP and BMAC (group A) and 76.2% (32/42 joints) in arthrodesis without highly porous β-TCP and BMAC (group B; P = .048).

**Conclusion:**

This study investigated the efficiency of highly porous β-TCP and BMAC to promote bony healing in midfoot joint arthrodesis. A significantly higher union rate was shown when arthrodesis was performed with highly porous β-TCP and BMAC, compared with arthrodesis performed without them. We suggest that highly porous β-TCP and BMAC can be a viable and effective adjunct to the fixation in midfoot joint arthrodesis.

**Level of Evidence:**

Level III: Retrospective comparative analysis

“This study investigated the efficiency of highly porous β-TCP and BMAC to promote bony healing in midfoot joint arthrodesis. A significantly higher union rate was shown when arthrodesis was performed with highly porous β-TCP and BMAC compared with arthrodesis without them . . .”

## Introduction

One of the most common and devastating complications following arthrodesis procedures is nonunion. Haddad et al^1^ reported that the rate of nonunion following primary arthrodesis of the foot or ankle approximated 10%. However, it can be higher in certain populations with risk factors, such as diabetes mellitus, smoking, and high body mass index (BMI).^2,3^ The rate of the osseous union can be affected by the type of surgical fixation technique and the absence of bone graft.^4-6^

Many different types of bone grafts and bone substitutes have been used to promote osseous fusion in foot and ankle arthrodesis. Autogenous bone graft from the iliac crest, proximal tibia, and calcaneus has been used traditionally and considered as the ideal bone grafting material.^7-9^ But, complications at the donor site have been reported, such as chronic pain, infection, nerve injury, blood loss, and seroma.^10-15^ Furthermore, the harvesting procedure itself takes time and cost, and the quantity and quality of the bone graft is variable depending on patients.^16^ Concerning these limitations, biologic bone substitutes have been sought and tried in arthrodesis procedures.^17-21^

Highly porous β-tricalcium phosphate (β-TCP) is one of the safe and effective alternatives to the autogenous bone graft. This biomaterial is highly biocompatible when implanted in bone, and histologic studies showed it was resorbable within 6 to 9 months.^22^ One of the main characteristics of highly porous β-TCP is to be commercially available as scaffolds with a macroporosity that provides osteoconduction and can reach 85% of the total mass of the grafted material.^23^ Its biocompatibility, bioresorption, and osteoconductive properties from the recipient’s bone make it a reliable material for the bone graft.^23^ Several studies reported the effect of β-TCP on promoting bone healing in foot and ankle arthrodesis when combined with the recombinant human Platelet-Derived Growth Factor BB (rhPDGF-BB).^18,19,21^

Besides suitable bone alternatives, bone marrow aspirate concentrate (BMAC) has been successfully used for bony healing, and it has gained popularity as an augmentation in the broad spectrum of foot and ankle areas.^24-27^ Mesenchymal stem cells (MSCs) within BMAC have the potential with the capability to differentiate into osteogenic progenitors, which will play a prominent therapeutic role in soft tissue and bony healing.^28-30^ Furthermore, many growth factors, including BMPs (bone morphogenetic proteins), platelet-derived growth factor, and transforming growth factor-β are contained in BMAC that can aid in bony healing.^27,31,32^

We hypothesized that BMAC could stimulate bony healing by promoting osteoinduction and osteogenesis when added on highly porous β-TCP, an osteoconductive scaffold. The purpose of this study is to investigate the efficiency of highly porous β-TCP with BMAC in midfoot joint arthrodesis.

## Methods

After obtaining approval from our institutional review board, a retrospective review was performed on patients who underwent midfoot joint arthrodesis, using compression screw, from January 2014 to May 2019. The inclusion criteria were (1) arthrodesis of a single or multiple midfoot joints using compression screws, (2) at least 18 years of age, and (3) follow-up until union or nonunion could be determined. The midfoot joints included tarsometatarsal (TMT), medial naviculocuneiform (NC), middle NC, lateral NC, and intercuneiform joints in this study. The exclusion criteria were (1) arthrodesis using different fixation constructs other than compression screws, (2) different types of bone grafts or bone substitutes other than highly porous β-TCP, (3) revision surgery, (4) diabetic patients whose hemoglobin A1C level is greater than 7.5, and (5) Charcot arthropathy.

Demographic and operative data, including age, sex, BMI, smoking, etiology of arthritis, comorbidities such as diabetes mellitus and rheumatoid arthritis, number of joints fixed, use of bone graft or bone substitute, and postoperative midfoot anatomic alignment in the 2 groups were compared.

The primary outcome in our study was radiographic evidence of complete union in patients who underwent midfoot joint arthrodesis using compression screws with or without highly porous β-TCP and BMAC. Weight-bearing foot X-ray was performed 6 weeks after the surgery to assess bony healing of the operative midfoot joints. Radiographic healing was defined as bridging bone across 3 cortices, and a nonunion was defined as a lack of radiographic union at 6 months without progression on sequential radiographs.^33-35^

The postoperative foot alignment was also evaluated as nonanatomic alignment following midfoot joint arthrodesis was a significant predictor of nonunion.^4^ Anteroposterior (AP) view confirmed the alignment between the medial edge of the second metatarsal and the medial edge of the middle cuneiform bone. Internal oblique view confirmed the alignment between the medial border of the third metatarsal and the medial border of lateral cuneiform and between the medial border of the fourth metatarsal and the medial border of the cuboid bone; the talo-first metatarsal angle (Meary’s angle) was measured using lateral view. Lateral foot alignment was considered anatomic if the Meary’s angle was between 4° and –4°. If any view of postoperative weight-bearing radiographic parameters was not anatomic, it was considered to be nonanatomic alignment. In addition, any postoperative complications including hardware irritation and infection were reviewed. All of these outcomes were compared between the 2 groups.

**Figure 1.**
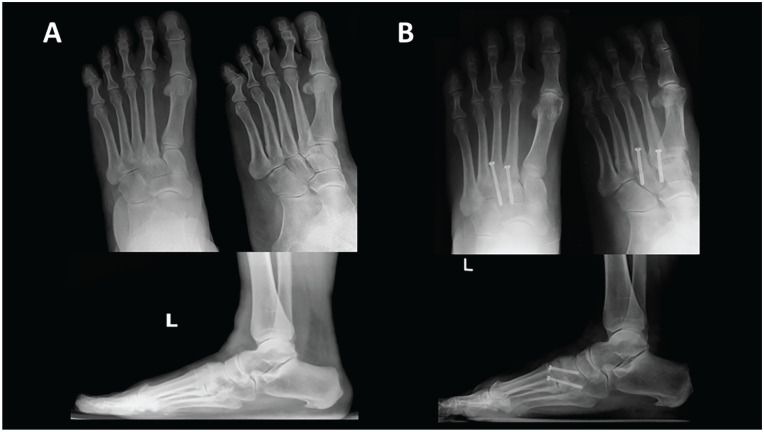
(A) Preoperative anteroposterior (AP), lateral, and oblique radiographs of an arthritis midfoot (second and third tarsometatarsal joint). (B) Sixteen-week postoperative AP, lateral and oblique radiographs from the same patient—second and third TMT joints arthrodesis using compression screws. Abbreviation: TMT, tarsometatarsal.

In this section, we describe the operative procedure involving midfoot joint arthrodesis using compression screws. The surgical technique and postoperative care for this study were also described in our previous study that compared 4 different fixation constructions for midfoot arthrodesis.^36^ All patients underwent surgery by one of 3 orthopedic foot and ankle surgeons at a single academic institution. They were in the supine position and under tourniquet control. A longitudinal medial incision in the interval between the anterior tibialis tendon and the posterior tibialis tendon was used for first TMT joint or medial NC joint, or a longitudinal dorsal incision was made for second TMT joint, third TMT joint, or middle/lateral NC joint. A single or dual incision was used, depending on the affected joints. The affected articular surfaces were debrided and resected using osteotomes, curettes, and sometimes resurfacing burs. Each joint was irrigated and drilled using multiple passes of a drill bit. Highly porous β-TCP (Vitoss; Stryker, Kalamazoo, Michigan) with BMAC (Harvest Technologies, Plymouth, Massachusetts) were used for bone graft. For the BMAC procedure, iliac crest was prepped and draped in the usual sterile fashion. A small nick was made in the skin over the anterior iliac crest about 3 cm posterior to the anterior superior iliac spine. Dissection was then carried bluntly down to bone and the needle was introduced and placed on the iliac crest and then introduced into the intramedullary cavity. Then aspiration was performed for 30 mL of bone marrow aspirate. This was done by alternating the position of the needle approximately every 5 mL. The aspirate was then filtered and then passed off for concentration. The total amount of the final BMAC production was 4 mL. It was mixed with β-TCP before the bone graft. The amount of β-TCP was determined between 2.5 cc and 5.0 cc by the number of joints fused. The graft of β-TCP and BMAC was inserted in the joint before the screw fixation. The decision to use bone graft or bone substitute was at the operating surgeon’s discretion. Once the decision was made to use the bone substitute for the patient, it was applied to all joints involved in the arthrodesis procedure, especially if defects or gaps were observed intraoperatively. The joints were manipulated to achieve plantigrade alignment of the foot. Then, the compression and fixation of the joints were achieved using partially threaded screws (Figure 1). Postoperatively, a well-padded short leg splint was applied. It was converted to a short leg non-weight-bearing cast at 2 weeks postoperatively. Non-weight-bearing immobilization was maintained for 6 weeks from the day of surgery. The cast was removed and a controlled ankle movement (CAM) boot was applied at 6 weeks postoperative follow-up visit. The patients were advised to bear weight in the CAM boot as tolerated. Radiographic evaluation of the union was performed between 6 weeks and 12 weeks postoperative follow-up visit. If there were any clinical symptoms such as pain or swelling, or radiological evidence of nonunion, then a further period of non-weight-bearing was recommended.

The 2 groups were compared using the Student *t* test for continuous variables and the χ^2^ test or the Fisher exact test for categorical variables. The level of statistical significance was set as *P* < .05. All statistical analyses were performed with SPSS software (Version 21.0; IBM, Armonk, New York). All normally distributed data are listed as mean and standard deviation with range; all non-normally distributed data are listed as median with range.

## Results

A total of 44 patients (46 feet) were included in this analysis. In our previous original study,^36^ 53 patients (55 feet) including 106 joints were included in the group of isolated compression screw fixation. Nine patients (9 feet) including 17 joints were excluded for this study as a different type of bone substitute other than highly porous β-TCP, which was the rhPDGF-BB, was utilized. The cohort was then stratified into 2 groups: 25 patients (26 feet) in arthrodesis with highly porous β-TCP and BMAC (group A) and 19 patients (20 feet) in arthrodesis without highly porous β-TCP and BMAC (group B). In our previous original study,^36^ 53 patients (55 feet) including 106 joints were included in the group of isolated compression screw fixation. Nine patients (9 feet), including 17 joints, were excluded for this study as a different type of bone substitute other than highly porous β-TCP was utilized, which was the rhPDGF-BB. The mean age of the whole cohort in this study was 59.6 ± 9.7 (range, 25-78) years. The overall BMI was 30.0 ± 6.6 (range, 19.8-48.7) on average. The follow-up period was 54.3 (range, 6.0-269.3) weeks. There were 8 (17.4%) men and 38 (82.6%) women. The demographic comparison between the 2 groups is shown in Table 1. There was no significant difference in demographics between the 2 groups.

**Table 1.** Comparison of Demographic Variables Between the 2 Groups.

Variables	Arthrodesis with bone substitute and BMAC (group A)	Arthrodesis without bone substitute and BMAC (group B)	*P* value
Patients (feet), n	25 (26)	19 (20)	
Joints, n			
Overall	47	42	
First tarsometatarsal (TMT)	3	9	
Second TMT	18	17	
Third TMT	18	13	
Medial naviculocuneiform (NC)	7	2	
Middle NC	0	0	
Lateral NC	0	0	
Intercuneiform	1	1	
Laterality, n (%)			.938
Right	12 (46.2)	9 (45.0)	
Left	14 (53.8)	11 (55.0)	
Sex, n (%)			>.999
Male	5 (19.2)	3 (15.0)	
Female	21 (80.8)	17 (85.0)	
Age, years, mean ± SD (range)	60.0 ± 12.2 (25-78)	59.0 ± 5.1 (51-69)	.733
BMI, kg/m^2^, mean ± SD (range)	29.2 ± 6.3 (20.5-48.7)	31.0 ± 7.0 (19.8-44.4)	.372
Diabetes mellitus, n (%)	1 (3.8)	3 (15.0)	.303
Smoking, n (%)			.520
Current	2 (7.7)	2 (10.0)	
Never	19 (73.1)	11 (55.0)	
Former	5 (19.2)	7 (35.0)	
Rheumatoid arthritis, n (%)	3 (11.5)	1 (5.0)	.622
Etiology of arthritis, n (%)			.347
Degenerative	22 (84.6)	14 (70.0)	
Posttraumatic	4 (15.4)	5 (25.0)	
Inflammatory	0 (0.0)	1 (5.0)	
Postoperative midfoot anatomic alignment, n (%)			>.999
Yes	23 (88.5)	18 (90.0)	
No	3 (11.5)	2 (10.0)	
Follow-up, weeks: median (range)	61.7 (9.3-210.0)	43.6 (6.0-269.3)	.615

Abbreviations: BMAC, bone marrow aspirate concentrate; BMI, body mass index.

We found a significant difference in the union rate between the 2 groups (Table 2). Group A, arthrodesis with highly porous β-TCP and BMAC, showed a significantly higher union rate than group B, arthrodesis without highly porous β-TCP and BMAC: 43 out of 47 joints (91.5%) versus 32 out of 42 joints (76.2%; *P* = .048). The location of nonunion is shown in Table 3. Most nonunion occurred at multiple joints simultaneously in the same patient who underwent multiple joints arthrodesis in group B, that is, arthrodesis without highly porous β-TCP and BMAC. Nonunion occurred in 5 patients (10 out of 12 joints) in group B, and all of them underwent multiple joints arthrodesis. Four out of 5 patients had nonunion at all joints at the same time, but only 1 patient had nonunion at 1 joint out of 3 involved joints. Compared with group B, nonunion occurred in 3 patients (4 out of 5 joints) in group A, that is, arthrodesis with highly porous β-TCP and BMAC: 1 patient had nonunion at multiple joints simultaneously, another patient had nonunion at 1 joint out of 2 involved joints, and the third patient had nonunion from the single joint arthrodesis procedure, which was an isolated medial NC joint arthrodesis case.

**Table 2.** Comparison of the Union Rate Between the 2 Groups.

Characteristic	Arthrodesis with bone substitute and BMAC (group A)	Arthrodesis without bone substitute and BMAC (group B)	*P* value
Union, n (%)	43/47 (91.5)	32/42 (76.2)	**.048**

Abbreviation: BMAC, bone marrow aspirate concentrate. Boldfaced if the *P* value is less than 0.05.

**Table 3.** The Location of Nonunion in the 2 Groups.

Characteristic	Arthrodesis with bone substitute and BMAC (group A)	Arthrodesis without bone substitute and BMAC (group B)
Nonunion, n (%)		
Overall	4/47 (8.5)	10/42 (23.8)
First tarsometatarsal (TMT)	0 (0.0)	3 (30.0)
Second TMT	1 (25.0)	4 (40.0)
Third TMT	2 (50.0)	3 (30.0)
Medial naviculocuneiform (NC)	1 (25.0)	0 (0.0)
Middle NC	0 (0.0)	0 (0.0)
Lateral NC	0 (0.0)	0 (0.0)
Intercuneiform	0 (0.0)	0 (0.0)

Abbreviation: BMAC, bone marrow aspirate concentrate.

During the follow-up period, a total of 4 postoperative complications were reported in this study (Table 4). In group A, 2 patients developed hardware irritation and all of them underwent removal of hardware procedure. In group B, 1 patient had a superficial wound infection, which was resolved completely without further treatment. The other patient in group B developed a deep wound infection with dehiscence and it was managed with irrigation and debridement followed by vacuum-assisted wound closure therapy.

**Table 4.** The Rate of Postoperative Complications Between the 2 Groups.

Characteristic	Arthrodesis with bone substitute and BMAC (group A)	Arthrodesis without bone substitute and BMAC (group B)	*P* value
Complication, n (%)			.908
Overall	2/47 (4.3)	2/42 (4.5)	
Hardware irritation	2 (100.0)	0 (0.0)	
Superficial wound infection with dehiscence	0 (0.0)	1 (50.0)	
Deep wound infection	0 (0.0)	1 (50.0)	

Abbreviation: BMAC, bone marrow aspirate concentrate.

## Discussion

This retrospective study aimed to prove the efficiency of highly porous β-TCP and BMAC in midfoot joint arthrodesis by comparing the union rate between the 2 groups: arthrodesis with and without highly porous β-TCP and BMAC. The result of this study reveals that patients who underwent arthrodesis with highly porous β-TCP and BMAC achieved a significantly higher union rate than those without highly porous β-TCP and BMAC. Nonunion is one of the most significant and concerning orthopedic problems after any arthrodesis procedure, and the rate of nonunion following midfoot joint arthrodesis has been reported variously from 2.0% to 12%.^34,35,37-39^ It develops due to biologic impairment, mechanical factors, or both. Bone grafts and bone substitutes have been widely used in foot and ankle arthrodesis procedures as they provide a positive effect on overcoming the biologic impairment. Buda and his colleagues demonstrated a significant reduction in the nonunion rate in their TMT joint fusion study when they used autologous bone graft harvested from the iliac crest, tibia, or calcaneus.^4^ Besides autogenous bone graft, bone substitutes such as rhPDGF-BB with β-TCP or β-TCP alone have been used in foot and ankle arthrodesis or periodontic procedures in previous studies, showing their efficiency in bony healing.^17,19,23,40^ DiGiovanni et al^20^ reported a significant higher fusion rate of hindfoot and ankle arthrodesis in the patients whose joints had adequate graft fill (≥ 50%) compared with the patients whose joints did not have adequate graft fill (< 50%), regardless of type or origin of graft material. Loveland et al^40^ performed a case series, analyzing patients undergoing Charcot reconstructions utilizing rhPDGF-BB/β-TCP for joint fusion. They found rhPDGF-BB/β-TCP to be a safe and effective graft material, showing a high union rate as of 97.3% (217 out of 223 joints) even in high-risk patients such as Charcot neuroarthropathy.

Osteoinduction is defined as the ability to induce bone formation, and osteoconduction is the ability of the recipient bone cells to colonize the graft.^23^ Autogenous cancellous bone graft is considered one of the ideal bone grafts as it can provide an osteoconductive, osteoinductive, and osteogenic substrate for filling bone voids and augmenting bony healing. Especially, autogenous cancellous bone graft from the anterior iliac crest was known to have a higher concentration of progenitor cells, compared with distal/proximal tibia and calcaneus.^41^ However, autogenous bone graft does have drawbacks, such as donor site morbidities and variability of quantity and quality of the bone graft. Thus, many studies have searched for safer and more effective bone graft and bone substitute materials to overcome these limitations. Daniels et al reported that rhPDGF-BB is promising in its ability to stimulate bone growth, conferring osteoinductive properties. Furthermore, its effect on bony healing can be more powerful when combined with the osteoconductive properties of a β-TCP scaffold.^42,43^ Several previous studies also verified the noninferiority of bone substitutes, compared with autogenous or allogenous bone grafts in bony union and clinical outcomes. DiGiovanni and his colleagues reported that treatment with rhPDGF-BB/β-TCP resulted in comparable fusion rates, less pain, and fewer side effects as compared with treatment with autograft in patients requiring hindfoot or ankle arthrodesis.^43^ Jeon et al compared bone union progression using highly porous β-TCP granules or allogeneic bone chips in the gap created by medial opening-wedge high tibial osteotomy (MOWHTO). They reported that MOWHTO using highly porous β-TCP granules had faster new bone remodeling and less radiographic sclerosis at the osteotomy margin.^44^

There is no consensus on the gold standard bone graft material and whether biologic bone graft materials should be used alone or in combination. In this study, we used highly porous β-TCP combined with BMAC. Bone marrow contains MSCs and HSCs (hematopoietic stem cells), and both of them have the potential to differentiate into osteogenic progenitors. This differentiation to osteogenic cells is capable of forming osteogenic tissues. Furthermore, BMAC has been widespread and successfully used based on this benefit, especially for bone and soft tissue healing. Hernigou et al compared 86 patients who received BMAC injection with 86 matched diabetic patients who received autogenous iliac bone graft for the ankle fracture nonunion treatment. Treatment with BMAC promoted nonunion healing in 70 out of 86 diabetic patients (82.1%), whereas 53 out of 86 diabetic patients (62.3%) with autogenous iliac bone graft had healing.^45^ Kennedy et al used BMAC as a biological adjunct to the treatment of OCLs (osteochondral lesions) and showed good medium-term functional outcomes.^46,47^ Murawski and Kennedy performed a case series reporting the results from 26 patients who underwent percutaneous internal fixation with a screw system of a proximal fifth metatarsal (Jones) fracture and BMAC. Their results showed that 24 of their cohort achieved union without complication at a mean of 5 weeks after the surgery.^25^ As they did not have a control group, however, there is a limitation in their study to evaluate the benefit of BMAC in bony healing. Our study was a case comparative study and appropriately designed to assess the efficiency of BMAC in osseous healing compared with the control group. A couple of locations have been used for bone marrow aspiration: the vertebral bodies, the posterior iliac crest, the anterior iliac crest, the proximal tibia, and calcaneus. Although vertebral bodies have been known to have a higher quantity of osteogenic progenitor cells, compared with other locations such as iliac crests,^48^ it is not practical to perform aspiration from the vertebral bodies in non-spine surgery. A previous study demonstrated that the anterior iliac crest contained a significantly higher mean concentration of osteogenic progenitor cells compared with the distal tibia and calcaneus in the same patient.^41^ There is another study demonstrating that the cell yield from the anterior and posterior iliac crest was the same but twice as much as that obtained from the proximal tibia.^49^ Thus, we performed all BMAC procedures from the anterior iliac crest bone in this study.

Previous biomechanical studies and clinical studies compared rigidity among many different fixation constructs and implants. However, there is still a debate with regard to the superiority of one fixation device over the others.^4,5,50-54^ Most recently, Ettinger et al compared the rate of nonunion following TMT joint arthrodesis using the isolated screw with locking plate plus compression screw fixation. In their study, combined locking plate and compression screw fixation was associated with lower nonunion rates compared with isolated screw fixation.^5^ Especially for the first TMT joint, several studies reported a significant difference in the union rate according to the fixation construct. DeVries and his colleagues found a superior union rate when using a dorsal medial locking plate with or without lag screw compared with crossed screw constructs for patients with Lapidus procedure.^33^ Klos et al investigated the utility of fixation with a medial locking plate with compression screw versus a fixation with 2 crossed screws in their biomechanical study. Their results demonstrated that the plate with screw construct had significantly less movement during testing and more cycles to failure than the crossed screw fixation.^51^ This study is an extension of our previous study that compared 4 different fixation constructions for midfoot arthrodesis.^36^ Among the 4 different fixation strategies, namely, staple fixation, compression plate fixation, compression plate with lag screw fixation, and compression screw fixation, the isolated compression screw fixation was shown as one of the independent predictors of nonunion following midfoot arthrodesis. In our study, we included only the patients who underwent midfoot arthrodesis with isolated compression screw fixation to evaluate how much highly porous β-TCP with BMAC can increase the bony union rate. The union rate was relatively low as of 76.2% (32/42) when arthrodesis was performed without bone substitute. This result echoes the previous studies’ findings that fixation with isolated compression screw may be inferior to other fixation constructions regarding the union rate. However, it can be considered a good opportunity to verify the benefit of highly porous β-TCP and BMAC in bony healing following midfoot joint arthrodesis. The union rate went up to 91.5% (43/47) when arthrodesis was performed with highly porous β-TCP and BMAC in our study, proving the benefit of the bone substitute.

This study is not without limitations. First, this is a comparative radiographic study and did not obtain patients’ clinical outcomes. Second, plain radiographs were used to determine the nonunion although computer tomography (CT) imaging has been considered the gold standard for assessing the nonunion. Third, only 1 fixation construct was used in this study, which may affect the union rate. However, we avoided any confounding factors that may come from different fixation constructs with this study design. Fourth, the final decision to use bone graft or bone substitute was at the surgeon’s discretion although it was made based on the finding of the defect on the field and preoperative images, including X-ray and CT. We admit that it could create a selection bias, which might include more severe cases for group A, that is, arthrodesis with highly porous β-TCP and BMAC. However, even if group A had included more severe cases, the union rate in group A was significantly higher than in group B. This result can show the efficiency of highly porous β-TCP with BMAC in midfoot joint arthrodesis. Fifth, patients with a hemoglobin A1C level greater than 7.5 were excluded from this study. This suggests that our findings regarding the union rate following arthrodesis may pertain primarily to a relatively optimized patient population in terms of diabetes management. Nevertheless, we still believe our results are noteworthy as we advise against performing midfoot joint arthrodesis for individuals whose diabetes is not adequately controlled, owing to the potential risks associated with postoperative complications. Finally, there are some limitations inherent to the retrospective nature of the study.

This study investigated the efficiency of highly porous β-TCP and BMAC to promote bony healing in midfoot joint arthrodesis. A significantly higher union rate was shown when arthrodesis was performed with highly porous β-TCP and BMAC compared with arthrodesis without them, suggesting that highly porous β-TCP and BMAC can be a viable and effective adjunct to the fixation in midfoot joint arthrodesis.
